# Perceiving jittering self-motion in a field of lollipops from ages 4 to 95

**DOI:** 10.1371/journal.pone.0241087

**Published:** 2020-10-23

**Authors:** Nils-Alexander Bury, Michael R. Jenkin, Robert S. Allison, Laurence R. Harris

**Affiliations:** 1 Centre for Vision Research, York University, Toronto, ON, Canada; 2 Dept. of Psychology, York University, Toronto, ON, Canada; 3 Institute of Visual Computing, Hochschule Bonn-Rhein-Sieg, Sankt Augustin, Germany; 4 Dept. of Electrical Engineering and Computer Science, York University, Toronto, ON, Canada; University of Minnesota, UNITED STATES

## Abstract

An internal model of self-motion provides a fundamental basis for action in our daily lives, yet little is known about its development. The ability to control self-motion develops in youth and often deteriorates with advanced age. Self-motion generates relative motion between the viewer and the environment. Thus, the smoothness of the visual motion created will vary as control improves. Here, we study the influence of the smoothness of visually simulated self-motion on an observer’s ability to judge how far they have travelled over a wide range of ages. Previous studies were typically highly controlled and concentrated on university students. But are such populations representative of the general public? And are there developmental and sex effects? Here, estimates of distance travelled (visual odometry) during visually induced self-motion were obtained from 466 participants drawn from visitors to a public science museum. Participants were presented with visual motion that simulated forward linear self-motion through a field of lollipops using a head-mounted virtual reality display. They judged the distance of their simulated motion by indicating when they had reached the position of a previously presented target. The simulated visual motion was presented with or without horizontal or vertical sinusoidal jitter. Participants’ responses indicated that they felt they travelled further in the presence of vertical jitter. The effectiveness of the display increased with age over all jitter conditions. The estimated time for participants to feel that they had started to move also increased slightly with age. There were no differences between the sexes. These results suggest that age should be taken into account when generating motion in a virtual reality environment. Citizen science studies like this can provide a unique and valuable insight into perceptual processes in a truly representative sample of people.

## Introduction

Judgement and control of one’s own self-motion is a fundamental skill for navigating successfully through the world. Humans typically learn to locomote independently at a young age with onset of independent walking typically occurring very early in the second year of life after a period of pre-walking locomotion [1]. Control and coordination of self-motion continues to develop and mature through childhood. Part of this development is the effective use of multimodal sensory information in the control of locomotor behavior and balance. Vision is a key input that both children and adults use to monitor their self-motion and stability. When a person is standing stationary and a specially constructed swinging room is translated relative to them, they tend to feel a compelling sense of self-motion and make significant postural sway adjustments [2]. Infants (aged 13–16 months) make more substantial postural responses to motion of their surroundings, often falling over [3].

As well as eliciting postural responses, when a large visual display moves with respect to an observer a compelling sense of self-motion can be produced. This phenomenon is known as vection. Many people have had the everyday experience of vection when on a stationary train but observing a moving train on the next track. While vection is illusory in situations such as the train illusion described above or in the case of Lishman and Lee’s [2] moving room, visual motion normally informs us of our movements through real environments. Vection also seems to develop as we mature and refine our abilities to control our self-motion. Shirai et al. [4] reported that vection onset was more rapid and vection was stronger for school children (aged 6–12 years) than for young adults. This is consistent with postural sway studies suggesting that children overly emphasize vision in self-motion perception. While postural sway seems to resemble the adult pattern by 7–10 years [5–7], Shirai and colleagues [8, 9] found that older children (13–15 years) also experienced stronger vection than young adults (20–22 years). This is consistent with other evidence that while sway responses to visual motion reach adult levels early in childhood, sensory integration in the face of cue conflict continues to develop [10] as does sensitivity to vection [11].

The functional role of the conscious perception of vection is not entirely clear despite self-awareness of one’s own motion being phenomenologically salient and consistent [12]. One possible role is in the monitoring and control of our motion to achieve locomotor goals. For instance, humans can judge the linear distance they have travelled or the degree that they have turned, a task known as spatial updating or visual odometry, by vision alone [13, 14]. While vection is not strictly necessary for visual spatial updating, performance seems to improve under conditions likely to elicit vection [15, 16] and vection alone can produce updating of the perceived direction of environmental features [17]. More directly, Riecke et al. [18] reported that pointing-based measures of spatial updating improved when the display elicited illusory self-motion. As with postural control and vection, the ability to perform spatial updating appears to develop through childhood. Children as young as 3 years old appear to be able to spatially update the location of a hidden object following a self-produced 180° change in viewpoint [19]. Petrini et al. [20] had adults and 10-to-11-year-old children reproduce a path by replicating it through blind walking (walking to reproduce the path in the dark). They experienced the path by being guided along it in the dark (physical motion), being guided along it in a lit room (physical + visual motion) or viewed a movie of travel down the path (visual motion). Interestingly, children showed evidence of combining visual and physical motion cues when both were available, but adults did not. One possible explanation suggested by the authors is that children may be more visually dominated and might not selectively discount visual information.

Furthermore, there may also be significant differences in the way self-motion is perceived by males and females. Although both are exposed to the same sensory inputs as they move around the world, females generally rely more on external visual cues in many spatial orientation tasks [21, 22] and are less susceptible to visual-vestibular conflict [23]. So far, there are mixed findings on sex differences and vection perception. Some studies find that females rate vection as more convincing than males [24] and have shorter onset latencies for circular vection [25]. However, other studies have failed to find differences in latency [24], and few studies have looked at sex differences in the perception of linear vection. There have also been both anecdotal reports of gender biases in cybersickness as well as controlled experiments [26] yet the question is not fully addressed (see [27]). In the present experiment we study path integration from visual self-motion in people over a wide age range and of both sexes using visual odometry to estimate how much visual motion is needed to evoke the perception of travelling through a specific distance [14, 28, 29].

Except when traveling in a vehicle, the visual motion produced by natural self-motion is not usually smooth but includes components produced by the bob, sway, lunge and rotation of our heads [30–33]. Adding these movements to a visual simulation of self-motion might be expected to make the signal corresponding to overall forward self-motion more difficult to extract because the additional components that might potentially act as noise. Adding such time-varying simulated head motions would also be expected to produce sustained visual-vestibular cue conflict. Contrary to these expectations of impairing visual self-motion, adding such jitter to self-motion simulations increases the likelihood and magnitude of vection [34]; see [35] for a review. Adding viewpoint jitter to visually simulated self-motion also improves the perception of distance travelled [36, 37]. The underlying reason that jittering flow increases percepts of self-motion is currently unclear [35, 38, 39]. Palmisano et al. [35] outlined several possible explanations including that jitter might improve the perception of 3D layout or scene rigidity, reduce motion adaptation, increase retinal slip, indirectly suppress (or stimulate) vestibular cortical areas, or provide a more ecological stimulus better matched to self-motion processing. As these authors point out, each of these explanations has limitations. Regardless of the underlying cause of the jitter effect, exposure and experience of smooth and jittering optic flow could be expected to vary over the lifespan. Children develop the ability to stabilize their head in space during increasingly difficult locomotion tasks as they mature, being able to do so on flat ground typically between 3 and 6 years of age, on narrow paths by 7 or 8, and by adulthood the walker typically and effectively compensates for lateral body motions [40]. Thus, the amount of viewpoint jitter expected as a consequence of everyday walking will vary with age. To our knowledge, the effect of age on the jitter enhancement of vection has never been explored. In the present experiments we do so using visual odometry.

The vast majority of self-motion perception experiments take place in highly controlled environments and with specially selected participants. Although such studies are critical to the advancement of science, the constraints of such studies can limit their application to the wider population. One critical issue with highly controlled experiments is that they can introduce an unintended bias in the results as the participant pool may be biased in one way or another relative to the general population. Scientific studies on self-motion, for example, often take place in universities and draw their participant pool from the student population. Young, healthy and soon to be better educated individuals may not necessarily reflect properties of the entire population. Although such populations may introduce biases, they also come with a number of positive properties. They are often a captive audience in that they are willing to engage in the study as there is some direct reward such as course credit for participating in a study at a university. In addition, such participants are often motivated in that they perceive that not performing well in the study may impact their “participation grade”. A number of studies have sought to characterize potential participant pool biases in perception experiments and psychology more broadly. Henrich et al. [41] observed, for example, that characteristics of university students from western societies are not necessarily representative of humans more broadly. Sharp et al. [42] examined the practice of providing course credit for participating in psychology experiments and perhaps not surprisingly found significant differences in student participation depending on the amount of course credit they were awarded. Philips et al. [43] in arguing for the benefit of ‘citizen science’, found no significant difference between altruistic participant performance and the performance of participants who received course credit. But they also observed that low-skill participant biases, as well as high-skill engaged participant biases, should be controlled for when assessing citizen scientist participants’ data.

In order to investigate the potential for population bias in earlier works related to the perception of linear self-motion with and without induced jitter, and to explore the potential for sex and developmental effects that can be difficult to explore within an undergraduate population, we adapted a protocol that has been used successfully in a controlled laboratory setting for use in a public space. A typical controlled laboratory experiment, such as that used in Redlick et al. [14], is to perceptually isolate the participant from the external environment using some form of virtual display, and then to present a reasonably large number of individual trials to probe the question under consideration. In a traditional experiment, stimuli are highly controlled (Gaussian blobs, random textures, random flickering lines, etc.) which provides good control but does not necessarily engage the participant with the simulation. It is typical to have a participant participate in large numbers of trials, perhaps collected over several sessions. Such an approach is not possible in the citizen participants model used here. Such participants cannot be expected to sit still for a long and repetitive data collection session. Furthermore, while not sacrificing the scientific question being asked, the display itself must be engaging in order to hold the participants’ attention. For example, replacing random flickering lines with a field of flickering lollipops to define motion cues and to replace a buzzer indicating that the participant had moved too far with a cartoon space bunny who interacted with the participants. Finally, as the intent was to collect data from a large number of participants (in the study reported here over 1,100 individuals experienced the experiment) it is necessary to deploy the experiment using compact, commodity hardware that can be easily maintained and disinfected between participants. A key problem in citizen science studies is reducing the set of conditions to an absolute minimum and trade off the ability to capture detailed properties of a small number of participants with more gross effects of a vastly larger number of participants.

## Methods

### Participants

**Fig 1:**
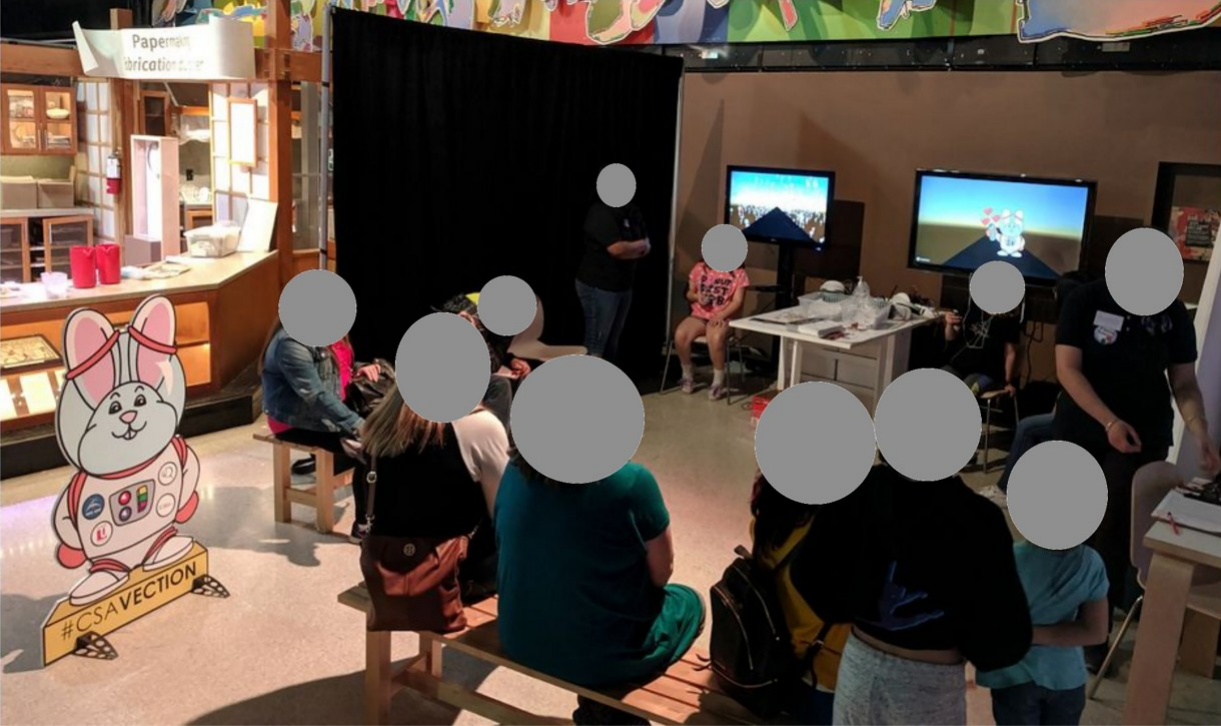
Experimental data collection at the Ontario Science Centre. Data were collected as part of the Summer of Space event over the summer of 2019. During the experiment visitors sat in upright chairs wearing earphones that provided audio instructions. Head tracking was disabled, and participants were encouraged to maintain the head in a level and facing forward direction. The participants’ view was mirrored on the screens behind them and the large ‘space bunny’ was presented in the real world to provide a standard size when the bunny was used to indicate the target distance in VR. Up to two participants were run simultaneously. All personal identifying features have been obscured.

Participants were recruited as part of a public science demonstration (the Summer of Space) held at the Ontario Science Centre in Toronto, Canada. Data were collected on weekends from May 25, 2019 to June 30, 2019. All visitors to our presentation were encouraged to experience the effect of vection in virtual reality but experimental data were only collected from those willing and able to provide informed consent. Table 1 shows general attendance to the Ontario Science Centre as well as the number of visitors to our demonstration over this period. As a consequence of the need to capture an informed consent to participate in this study only visitors between the ages of 4–15 years who were accompanied by a parent/guardian or were 16 years of age or older, had their data captured (the participants). These participants varied in age from 4 to 95 years old. All participants or their guardians signed consent forms and had the contents of the form clearly explained to them. Of the 1,183 visitors who experienced the experiment, 1,179 completed the questionnaire. However, only 871 agreed to have the full extent of their data analyzed. All experiments were approved by the York ethics board and were run in accordance with the Treaty of Helsinki. Participants received no financial compensation but all visitors to the booth received a sticker and a paper certificate for their help. The booth itself is shown in Fig 1. Two experimental sessions were run in parallel (see Fig 1).

**Table 1:** Visitors to the Ontario Science Centre and our event by date.

Week	Saturday Attendance	Saturday Visitors	Sunday Attendance	Sunday Visitors
13-May-2019	3063	101	3000	74
20-May-2019	5134	77	1305	50
27-May-2019	2796	107	1865	59
03-Jun-2019	1607	97	1279	85
10-Jun-2019	2962	117	1761	64
17-Jun-2019	1844	96	1814	71
24-Jun-2019	2300	104	2443	81

There was a total of 1,183 visitors to the exhibit. From these 1,183 visitors 871 met the ethical requirements to be considered as candidate participants and of these 466 met the performance requirements to be treated as participants and have their data analyzed.

### Visual display

Stimuli were presented in stereo on a Lenovo Mirage Solo HMD (5.5” QHD 2560x1440 LCD display refreshed at 75hz). Input was captured through the standard input device associated with the HMD and stimuli were rendered in Unity using the onboard Qualcomm Snapdragon 835 processor. Audio describing the experiment and relaying instructions were presented to the participants through earbuds connected to the HMD. These earbuds were also used to mute any external audio cues. The participants viewed the display while seated and were not tethered in any other way. During presentation of stimuli, head tracking was disabled resulting in a head-fixed display. Data were collected with participants sitting upright with their head facing forward (see Fig 1). Participant responses were collected using buttons on the Lenovo Bluetooth wand which was connected to the headset wirelessly. All computation including generation of the visual display, input capture, and response storage was performed using onboard computation on the HMD. Between participants these data were transmitted wirelessly to an external server, and the headset and input wand were sanitized.

**Fig 2:**
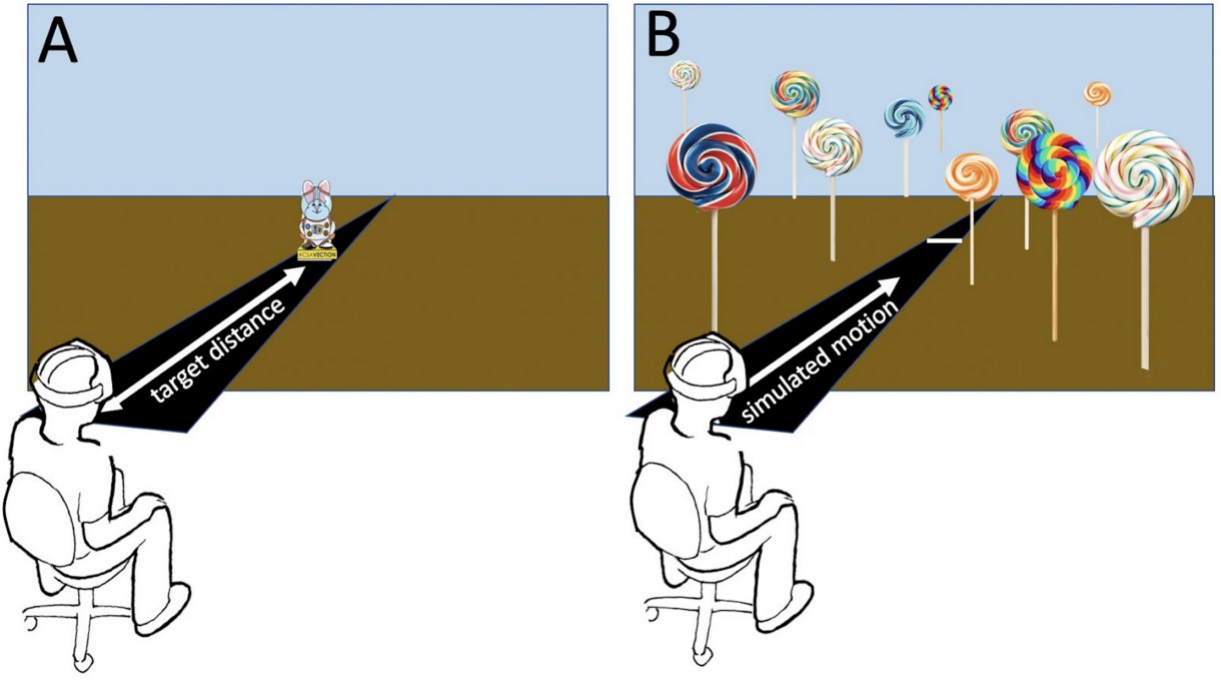
Participant’s view of the simulated environment. Before donning the HMD, participants were shown a cut-out full-size model of the target bunny visible in the photograph of Fig 1. In the VR display they saw the same bunny simulated at some distance away along a road (A). The bunny then disappeared, the areas outside the roadway became filled with colourful lollipops, and the participant was virtually moved down the road, signaled by the movement of the lollipops (B). Actual user displays were mirrored on monitors as shown in Fig 1.

Participants viewed a simulated environment with their head 1.1m above a simulated 3.3m wide black floor that stretched out in front of them to infinity. A field of randomly generated lollipops (see Fig 2) were presented in the space to the left, right and above the roadway. Lollipops were generated uniformly over the volume defined by the horizontal range -40m to +40m, vertical range -10m to +10m, and from the initial simulated position of the participant to 200m forward excluding the volume of space defined by the 3.3m wide black floor to a height of 3.2m. A total of 2,000 lollipops were generated within this volume facing towards the camera plane. Lollipops were generated and destroyed on a random schedule to prevent participants from tracking their position by following a single feature. On every frame lollipops were destroyed with a 1% probability. At each rendering frame, destroyed lollipops re-appeared at a random position with a 99% probability. Lollipops appeared with one of four different textures/patterns. A coloured skybox was displayed at infinity. Participants were presented with a stereo-rendered view and although the Lenovo HMD supports head tracking this feature was disabled during the experiment.

### Procedure

Upon completing the informed consent form and a short questionnaire presented on paper, participants were shown a large cardboard character (the space bunny shown in Fig 1) which they would later see presented simulated in the virtual environment. Participants were then directed to sit in a chair, assisted in donning the HMD along with its attached earbuds, and given a Bluetooth wand to hold. Each trial began with a flat stationary character—the space bunny—indicating a target distance along the road with the lollipops removed (see Fig 2A). An audio cue instructed the participant to ‘push the button when you get to me’, the space bunny then disappeared, and the visual field shown in Fig 2B was displayed. Immediately, the lollipops were moved past the participant simulating their movement down the roadway at 6m/s. This constant velocity motion continued until the participant pushed a button on the hand-held controller to indicate that they had reached the previously presented target distance. At this point the scene was extinguished and the next trial began. There was a total of 12 trials consisting of four target distances (10m, 20m, 30m, 40m) combined with three jitter conditions (horizontal, vertical, none)—one trial of each—that were presented in a randomized order. For jitter conditions the constant velocity motion down the hallway was augmented with horizontal or vertical sinusoidal motion at 1.5 Hz and an amplitude of 0.03m. These values were chosen to be consistent with head motions encountered during walking [31, 32]. If a participant had not responded by the time they had moved a simulated 150m horizontally through the virtual environment that data point was recorded as “missing” and the display moved to the next condition after the participant was presented with an audio cue that they had ‘not pushed the button’. Missed trials were not repeated. Upon completion of the 12 conditions the participant was instructed to remove the HMD and were given a sticker and certificate. Participants were also allowed to keep their earbuds as it was too complex to properly sanitize them for the next participant.

### Data analysis

For each jitter condition (none, horizontal, vertical), four target distances (10m, 20m, 30m, 40m) were presented. For all 871 candidate participants who completed the questionnaire and agreed to have their data analyzed, a straight line was fitted through the data points for each of the three jitter conditions following Redlick et al. [14].

Distancetravelled=targetdistance*slope+intercept (1)

The slope expresses how evocative the visual display was in generating vection. Slopes greater than one indicate that candidate participants under-estimated the simulated motion and needed more vision to evoke the sensation of reaching the previously viewed target’s position. Slopes less than one indicated that candidate participants over-estimated the simulated motion. The slope is thus the inverse of the Perceptual Gain defined as the perceived distance (the target distance) expressed a fraction of the amount of visual motion needed to evoke that perception (the distance travelled). A perceptual gain of unity is obtained if participants move accurately to where the target bunny was. More lollipop movement indicates a lower perception gain: Less lollipop movement indicates a higher perceptual gain.

Vection exhibits an onset latency [12] during which a participant does not register movement towards the target location. The intercept in Eq 1 provides an estimation of vection onset latency–the distance the lollipops moved before the participant felt themselves moving towards the target location. Since the velocity of travel was constant at 6m/s, the latency-of-onset is given by

Latencyofonset=intercept/velocityseconds (2)

**Fig 3:**
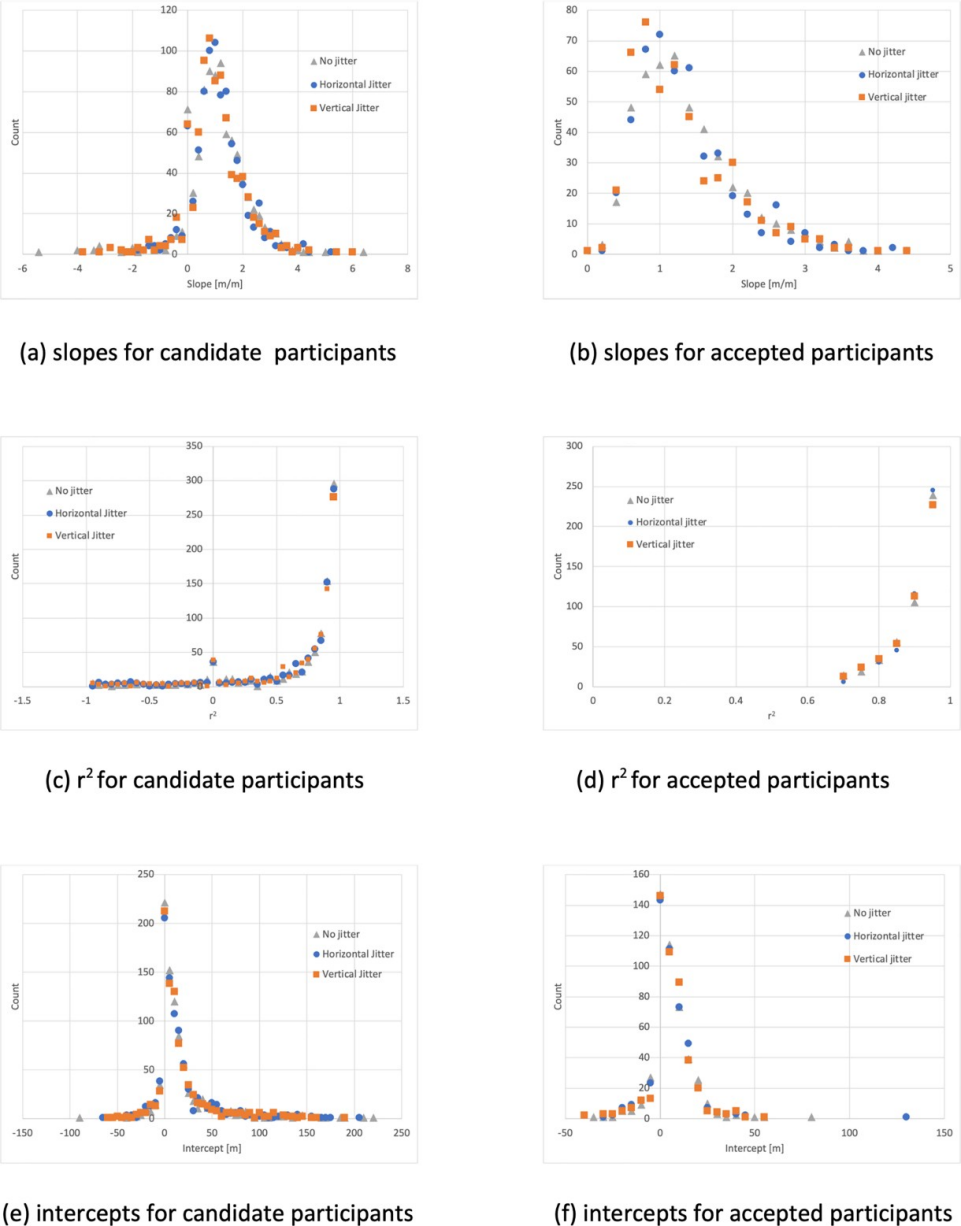
Regression lines. Regression lines were fitted to distance traveled vs target distance for all participants (left col) and to those who passed our acceptance criteria (right col) (see text). The distribution of slopes, is shown in a (all 871 participants) and b (466 accepted participants), the r^2^s (c, d) and the intercepts (e, f).

The distribution of the resulting slopes, intercepts and regression coefficients are plotted in the left column of Fig 3A, 3C and 3E.

To be included for further analysis candidate participants had to meet the following criteria. They needed to have:

1. provided responses for at least three of the four target distances for all three jitter conditions
2. an r^2^ value of 0.7 or above for all three jitter conditions
3. a slope greater than 0 for all three jitter conditions

**Fig 4:**
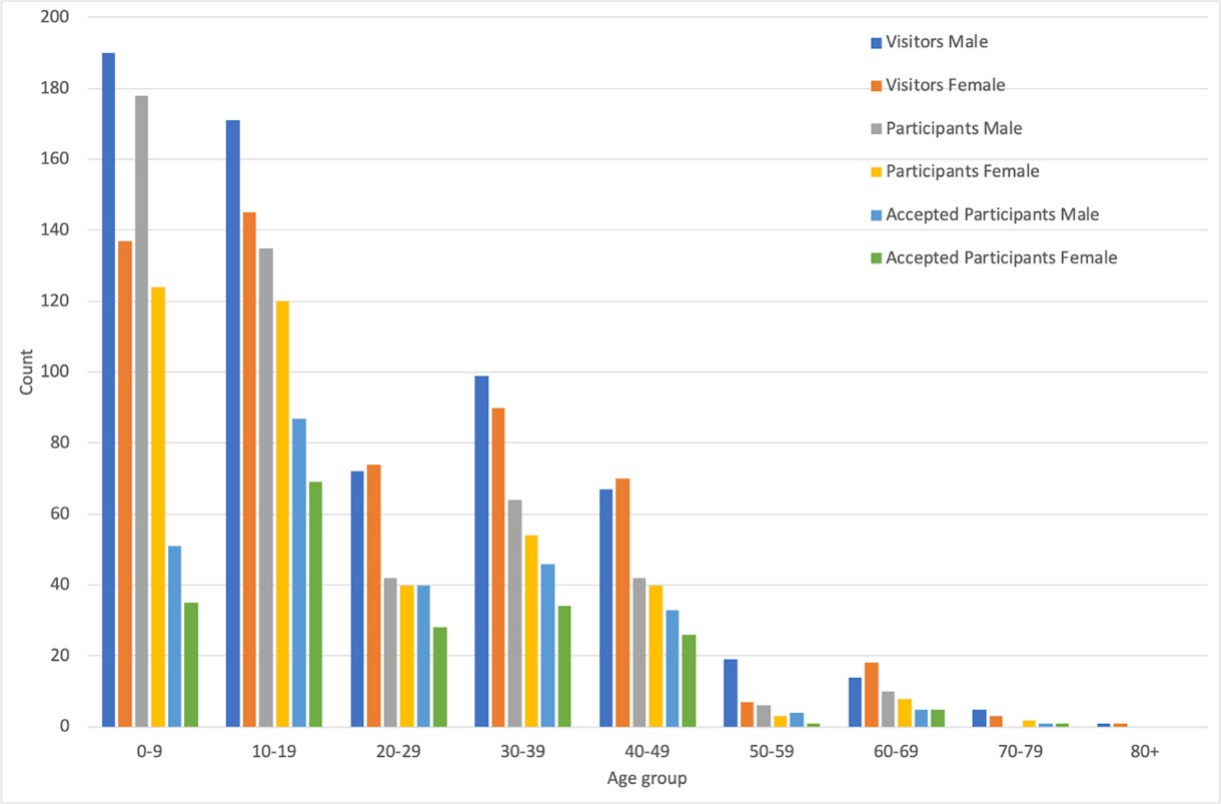
Numbers of people by age. Numbers of people in each of nine age ranges broken down by sex for total number of visitors (n = 1,183), participants (n = 871) and accepted participants (n = 466).

466 participants (267 males and 199 females) met all these criteria. This represents 53% of the candidate participants who could give informed consent to be studied and who had completed the questionnaire. Table 2 shows the numbers of candidate participants that met each of these criteria for each of the three jitter conditions. The distribution of slopes, intercepts and r^2^ for accepted participants’ data are shown in the right column of Fig 3B, 3D and 3F. Fig 4 summarizes the distribution of visitors, candidate participants and participants by age and sex. All of the analysis that follows utilizes this participant dataset and used SPSS for data analysis. The threshold for significance (alpha) was set at .05.

**Table 2:** Candidate participants who met performance criteria.

Jitter condition	3 or 4 valid distances	Valid slope	Valid r^2^
None	846	777	636
Horizontal	850	771	624
Vertical	843	768	624
Combined	822	681	466

Candidate participants had to provide three or four valid distance measurements for a given jitter condition. With three of four valid distance measurements a linear regression could be performed and slope and r^2^ values computed allowing the last two criteria (see methods) to be applied. Candidate participants had to meet the criteria for all three jitter conditions (the combined row in the table).

A mixed model ANOVA with a between participant factor of sex was performed to see if there was an effect of jitter or sex on the slope or intercept of the linear fit through the distance travelled plotted as a function of target distance.

## Results

### Main analysis

**Fig 5:**
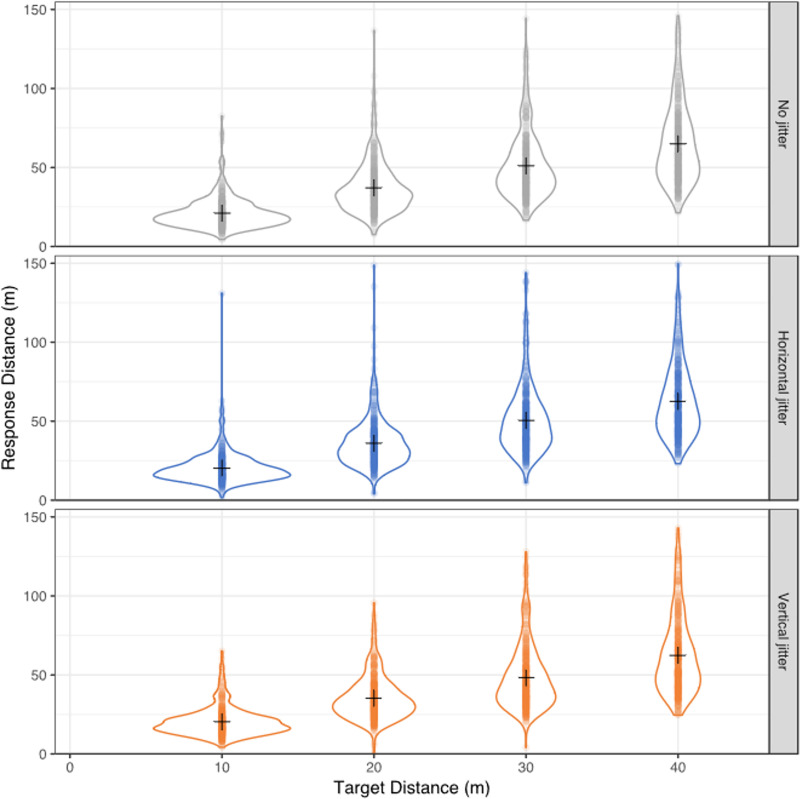
Distribution of participants’ responses. Distribution of participants’ responses as violin plots for each target distance (horizontal axis) and jitter condition (rows). Individual responses are shown as semi-transparent dots and the violin plot shows a mirrored representation of the kernel density estimate for the data as a function of travelled distance (vertical axis). Mean for each condition is indicated by a ‘+’ symbol.

Fig 5 shows the range of participant responses for each jitter condition and target distance. The dataset consisted of 267 males and 199 females. For the slope of the linear fit Mauchly’s test indicated that the assumption of sphericity had not been violated Χ^2^(2) = 0.097, p = 0.952, n.s. The results show an effect of jitter, F(2,928) = 3.020, p = 0.049. Post hoc t-tests using the Bonferroni correction showed a significant difference between the no jitter and vertical jitter conditions t(465) = 2.514, p = 0.036.

**Fig 6:**
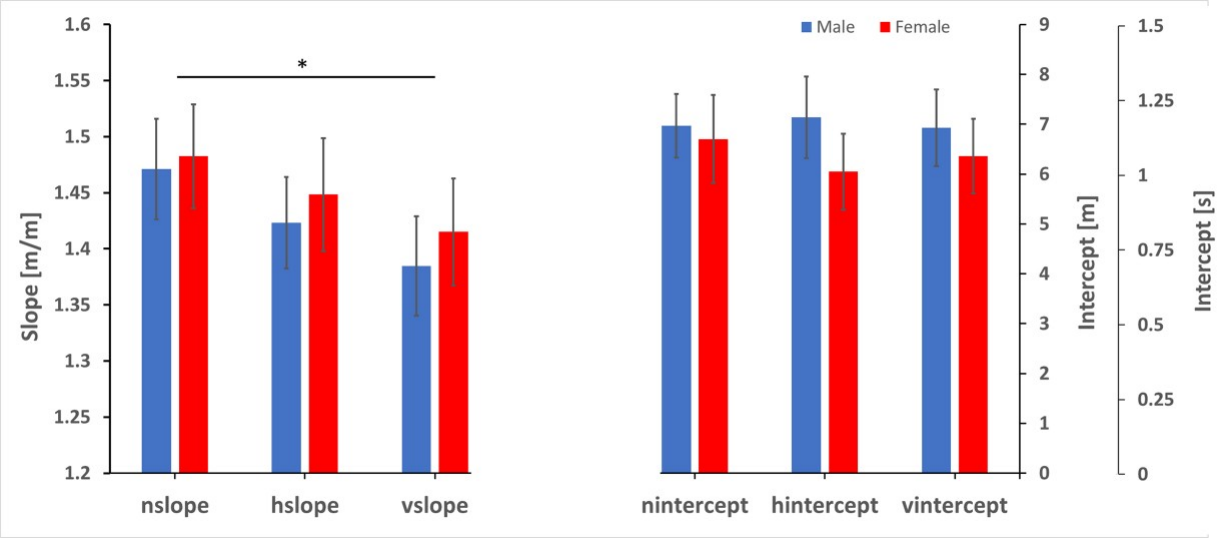
Slopes and intercepts. Slopes and intercepts for linear fits to the three vection conditions broken down by sex. (Blue males, red females). For the slope there is a significant difference between no jitter and vertical jitter conditions. For the intercept there was no significant difference between the conditions. nslope and nintercept refer to the no jitter condition, hslope and hintercept refer to the horizontal jitter condition while vslope and vintercept refer to the vertical jitter condition. The vertical axis for slopes is on the left while the one for intercepts is on the right. The intercept axis is given in m and the corresponding time in seconds.

Although the ANOVA showed that there was an effect of jitter, the effect size was small with a partial Eta squared of 0.006. The between participant factor of sex was not significant, F(2,928) = 0.049, p = 0.952 n.s; with a partial Eta squared < 0.001. For the intercept, Mauchly’s test indicated that the assumption of sphericity had been violated Χ^2^(2) = 6.148, p = 0.046. Degrees of freedom were corrected using Greenhouse-Geisser. There was no effect of condition on the intercept F(1.974,915.92) = 0.068, p = 0.935 n.s., with a partial Eta squared < 0.001. Nor was there an effect of sex, F(1.974,915.92) = 0.177, p = 0.835, n.s., with a partial Eta squared < 0.001. Fig 6 plots the mean slopes and intercepts for the three jitter conditions separated by sex. Adding vertical jitter enhances the efficacy of visual motion to induce vection meaning that less visual motion is required in the presence of vertical jitter to evoke the sensation of having moved through a particular target distance (lower slope, higher perceptual gain).

### Developmental effect

**Fig 7:**
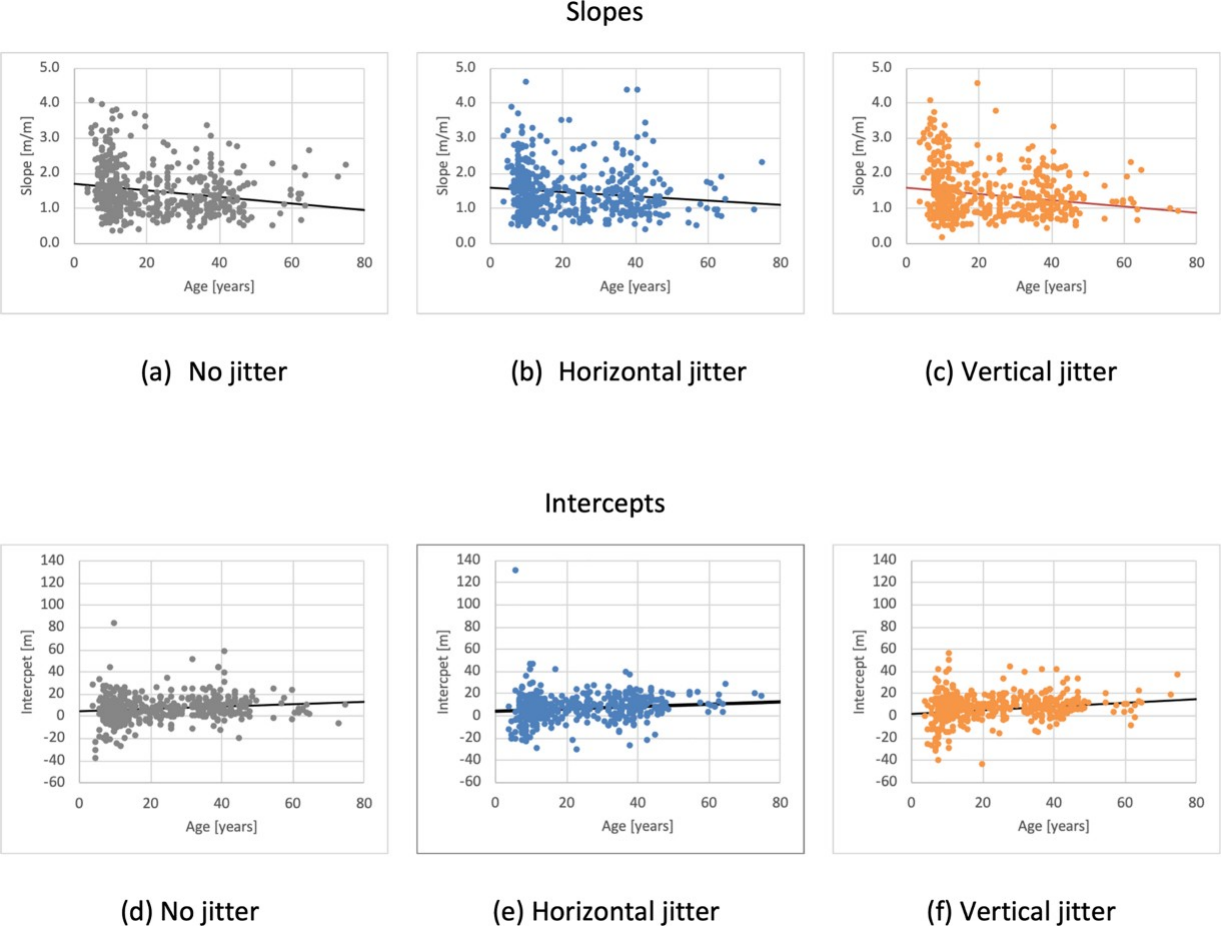
Linear regressions. Linear regressions through the model parameters as a function of age. Top row shows the effect of age on slopes for no jitter (a), horizontal jitter (b) and vertical jitter (c). Bottom row shows the effect of age on intercepts for the same three groups.

Given the unequal number of participants per age group (Fig 4) it was not practical to bin participants by age range for data analysis. Instead linear regression lines were fit to the raw model parameters as a function of participant age. Fig 7 plots linear regression lines through the model parameters for each jitter condition (slopes Fig 7A–7C; intercepts Fig 7D–7F) and Table 3 summarize the fit parameters and statistical properties of the fits. The linear regression of response slope for different jitter conditions as a function of age was significantly different than a model with no independent variable for no-jitter F(1,464) = 17.622, p<0.001 with an R^2^ of 0.037; for horizontal jitter; F(1,464) = 9.25, p = 0.002 with an R^2^ of 0.020; and for vertical jitter; F(1,464) = 17.188, p<0.001 with an R^2^ of 0.036. The response intercept for different conditions as a function of age was significantly different than a model with no independent variable for no-jitter F(1,464) = 10.954, p = 0.001 with an R^2^ of 0.023; for horizontal jitter; F(1,464) = 7.805, p = 0.005 with an R^2^ of 0.017; and for vertical jitter; F(1,464) = 20.343, p<0.001 with an R^2^ of 0.042. Although the ANOVAs showed a significant effect of age on both the slope and intercept, the effect size was small for all three jitter conditions for both the slope and intercept.

**Table 3:** Regression coefficients and statistical significance of fits.

	Unstandardized Coefficients	
JITTER	B	Std. Error
**Slope**		
None constant	1.684	.059
None age	-.009 delta slope/yr	.002
Horiz. constant	1.583	.058
Horiz. age	-.006 delta slope/yr	.002
Vertical constant	1.604	.059
Vertical age	-.009 delta slope/yr	.002
**Intercept**		
None constant	4.178 m	.961
	0.7s	
None age	.116 delta m/yr	.035
	0.019s/yr	
Horiz. constant	4.206 m	1.048
	0.7s	
Horiz. age	.106 delta m/yr	.038
	0.018s/yr	
Vertical constant	2.930 m	.990
	0.49s	
Vertical age	.162 delta m/yr	.036
	0.027s/yr	

“Constant” refers to plots of travelled distance vs target distance and “age” refers to plots of the parameters (slope or intercept) as a function of age. The slopes for the “constants” are unitless but for age are in slope change (delta) per year where a negative value indicates an increase in effectiveness of vision with age (shallower slopes, increased perceptual gain). The intercepts for the constants are given in metres or change (delta) in m/year. Intercepts can be converted to seconds by dividing by the speed (6 m/s). A positive intercept value indicates an increase with age.

Two mixed model ANOVAs were performed to see if there was an interaction between participant age and the jitter conditions. The first used the regression slopes between distance traveled vs target distance for each participant as the dependent variable and the second used the intercept. For the slope analysis, Mauchly’s test indicated that the assumption of sphericity had not been violated Χ^2^(2) = 0.004, p = 0.998 and the interaction between jitter and age was not significant F(118,812) = 0.892, p = 0.780, with an Eta squared value of 0.115. For the intercept analysis, Mauchly’s test indicated that the assumption of sphericity had been violated Χ^2^(2) = 6.782, p = 0.034, so Greenhouse-Geisser adjustments were made to the degrees of freedom. The interaction between jitter and age for the intercept was not significant F(116.072,798.736) = 0.870, p = 0.828 with a partial Eta squared of 0.112.

Coupled with the sign of the slope, these data indicate a small but significant decline in slope (increase in effectiveness of vision) as a function of age for all jitter conditions but no significant interaction between jitter condition and age. Thus, the older the participant the less simulated motion in VR was required in order to perceive that they had moved through a given distance regardless of the jitter condition. Similarly, there was a small but significant increase in the intercept as a function of age for all jitter conditions, but no significant interaction between jitter condition and age. Thus, the older the subject the more delayed their responses by about 0.02s/year (Table 3). The low R^2^ values capture the large amount of variability in the data which is evident in Fig 5.

## Discussion

Being able to properly interpret linear vection as self-motion is a key requirement for humans to function in the world. It allows for proper motor control for foot and hand placement and to judge time to contact. Normally self-motion is cued by a range of sensory information, not the least of which is the vestibular system that can provide strong cues as to linear accelerations acting on the body. Here we show that performance is equal between males and females and gets better with age and the addition of vertical jitter, although age does slightly slow performance.

Understanding the process of generating self-motion cues from vection is particularly important for virtual-reality based display systems where often motion cues are provided through visual cues alone. Virtual-reality based teleoperation systems and training systems, as examples, rely critically on visual cues for self-motion and understanding how these processes work over a range of different participants is critical if these systems are to be deployed generally. In virtual reality simulations it is often the case that the simulated motion is driven by software systems which are not sensitive to the sensory stimulation created during natural walking over terrain. A human’s head bobs and weaves as they move over the ground resulting in both horizontal and vertical jitter in the resulting visual input. Even in a vehicle, jitter is introduced when driving over rough roads. Is it desirable or necessary to simulate such jitter in a visual display in order to generate convincing vection? Previous work, as reviewed in [35], has demonstrated that added jitter enhances vection in controlled experiments with traditional participant pools. However, do these results generalize to a broader participant pool? And similarly, what role does age and sex play in the experience of vection?

### Effect of jitter

Adding coherent jitter to constant-velocity radial flow is known to enhance vection [34, 44]. Earlier studies have used relatively small participant groups. Here we have confirmed this effect over a large, naive population with ages ranging from 4 to 80 years. We found that adding horizontal or vertical oscillation to the virtual viewpoint during vection resulted in shallower slopes of travelled distance as a function of target distance. That is to say, less visual motion was required to induce the sensation of moving through a given distance when oscillation is added–a higher perceptual gain. Addition of jitter to simulations of self-motion has been found to promote vection in terms of shortened onset, increased duration and increased vection magnitude [34, 45, 46]. The average jitter enhancements found in the present study are much smaller than the approximately 40% of the average perceived speed increase from oscillation reported in another study [45].

Bossard and colleagues have found that visual odometry was more accurate when moderate frequency oscillation was added to the viewpoint than it was during smooth linear vection [36, 37]. The participants in Bossard’s studies tended to experience simulated travel distances that were smaller than target distances for target distances greater than 12m, indicating that they overestimated the distance travelled during vection. With jitter, this overestimation was smaller and thus distance estimates were closer to the target distances. In contrast, in the present experiment, on average our participants needed to move considerably farther in the virtual environment than the target distance, indicating that they underestimated the simulated distance travelled during optic flow (low perceptual gain). On average, with jitter, this underestimation was reduced, and responses were closer to the target distances. Thus, as in Bossard’s studies, presence of jittering compared to smooth flow improved the accuracy of the participants’ responses. However, in the case of Bossard et al. the pattern indicated that the participants experienced less self-motion in jittering compared to smooth flow, whereas in the current experiment the pattern was consistent with the observers experiencing more self-motion during jittering flow.

To date, studies on both the jitter effect and visual odometry have been laboratory based, relatively small numbers of academic participants (typically 20), and a restricted age range. The most directly comparable study was that of Bossard and Mestre [36] who also studied visual odometry and used 124 observers across their three experiments, predominantly university students in their early twenties. In the current study we had much broader participation from the general population and a wider age range. It is unclear whether the difference between the underestimation of visual motion found in the current study and the general overestimation found by Bossard is due to this difference in participant make-up or due to the differences in equipment, stimuli and environment (lab versus science museum). The jitter effect found by Bossard and colleagues is not consistent in direction with our current findings and the literature reviewed by Palmisano et al. [35], which has generally found that adding perspective jitter or oscillation produced larger magnitude and shorter latency vection responses. If this stronger vection sensation were integrated to arrive at a percept of distance travelled then, opposite to Bossard and Mestre’s findings but consistent with our current findings, observers would have felt that they reached the goal sooner in the presence of jitter compared to smooth flow. This difference in polarity is hard to reconcile but all studies of the jitter effect, including those of Bossard, have suggested that jitter ‘improves’ vection in the sense of producing responses that more closely match the simulated visual flow. However, in practice this advantage might be offset by the possibility that jitter could induce motion sickness [46].

### Lack of a sex effect

The high level of variability in participant responses makes it difficult to make strong conclusions about sex effects in this study. The question of whether sex has an effect of the perception of vection, either in response to radially expanding or contracting visual motion (simulating forwards or backwards translational self-motion) or laminar (simulating sideways or rotational self-motion), is not well addressed in the literature with studies coming down on both sides [see 47 for a review]. A key issue in the literature is the wide range of metrics used to measure vection. For example, the ability of a display to generate cybersickness—assessed either through questionnaires or physiological effects—has often been identified as being a strong indicator for a sex bias. Recent work [27] suggests that the use of head mounted display technology that is better suited to male physiology may be a contributor to this. However, none of our participants reported any sensation of cybersickness, which is probably due to the short test duration (<3min) and the lack of participant-induced head motion and tracking. Longer immersion in VR is associated with a high incidence of cybersickness and especially postural sway [48]. In a traditional self-motion experiment, such as [14], an individual participant is presented with multiple motion profiles (12 in [14]) with multiple target distances (4 in [14]) or approximately four times as long as the total VR presentation in the present study, which had 4 target distances and 3 jitter conditions. Although cybersickness can also occur when participants are head fixed (see [49]), head tracking and body motion are known to be contributing factors in terms of cybersickness and motion sickness (head tracking: [50], body motion: [49]), but the VR presented to the participants reduced the impact of both of these factors. In terms of measurement of vection directly, Wei et al. [51] in a rotational vection study found no sex effect in terms of vection onset as measured by participant self-reports, a finding in conflict with Darlington and Smith [25] and Kennedy et al. [52] who found that males exhibited substantially longer onset latencies. Here, with a large population of participants (267 males and 199 females) we find no sex effect for onset latency for linear self-motion.

### Developmental effect

The high level of variability in participant responses makes it difficult to make strong conclusions about developmental effects in this study. We had noted that as a person develops and improves their ability to stabilize their head during locomotion, their experiences with and expectations of perturbations during locomotion would also change. Contrary to our hypothesis of developmental modulation of the jitter enhancement of self-motion perception we did not find any evidence for an interaction between age and flow condition. In other words, we found a similar enhancement from jittering flow regardless of age. This null finding is hard to interpret but could indicate that the jitter effect is not tuned to the fine details of ecological jitter as others have argued [38] or that any developmental effect is small relative to inter-subject variability.

That being said, across all three jitter conditions there was a significant decline in slope (increase in perceptual gain) and a significant increase in intercept with age. A non-zero intercept can be attributed to a number of factors including software rendering and input detection delay, user reaction time delay, and a delay in the onset of vection. The positional offset can be converted into a temporal lag given the fixed simulated speed of self-motion used in this study (6m/s). The intercept (lag) increased linearly with age with a lag slope of 19 msec/year for no jitter, 18 msec/year for horizontal jitter, and 27 msec/year for vertical jitter. This is to be contrasted with simple reaction time latencies for visual stimuli increasing by approximately 5 msec/year, although this linear relationship was only measured for adults (>18yrs.) [53]. Thus, the increased latency with age is unlikely to be solely due to increased reaction time suggesting an increased delay in the generation of a useful level of vection that contributes to changes in the estimation of motion with age. Consistent with this conclusion, Shirai et al. [4, 8, 9] also found that vection latency was significantly shorter in children than young adults. The current study suggests a gradual increase in vection latency extending throughout the lifespan.

**Fig 8:**
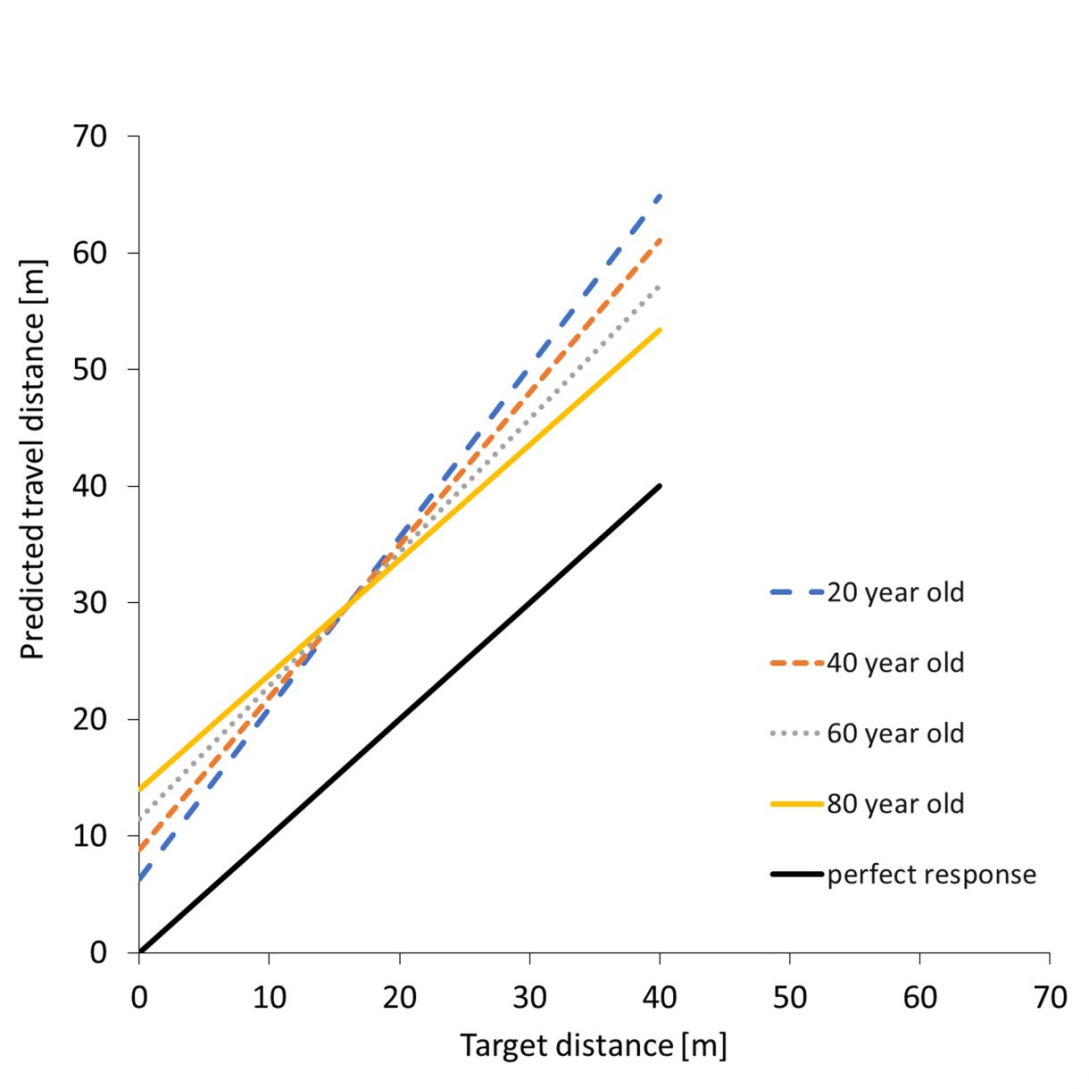
Predicted travel distance for different age groups. Participants were divided into groups by age and linear regressions fitted to their response distance plotted as a function of target distance. The resulting regression lines are shown here with the predicted distance (vertical axis) plotted as function of the target distance (horizontal axis) for four representative ages.

There was a significant decrease in the slope (distance travelled vs target distance) as a function of age. Together with the intercept, this allows us to predict the best linear fit for a participant of a given age as shown in Fig 8. Even with the increased delay in the onset of useful vection (slopes do not go through zero), we see a large change in the amount of useful vection provided by the stimulus, with all ages requiring a similar amount of visual motion to evoke a perceived travel distance of 17m and a modelled 20 year old participant requiring approximately 20% more visual motion than a modelled 80 year old participant to perceive they had travelled through a distance of 40m.

A number of studies have reported changes in perceived self-motion with age. For example, the ability to perceive the direction of self-motion (heading) is known to decline with age [54, 55]. It is unclear whether this imprecision in heading estimation might impact the perception of distance travelled. Furthermore, there is evidence that elderly people tend to rely more heavily on visual cues than other cues when estimating their motion [56], controlling their posture and movement [57–60], and their self-orientation [57]. Self-motion perception normally involves integrating multiple sensory cues including visual and vestibular cues. The processes underlying the combination of information across the senses (multisensory integration) are known to change with age [61, 62]. Older adults tend to exhibit heightened multisensory integration compared to younger adults [63–65]. Heightened multisensory integration, however, does not always lead to performance benefits and may even result in decrements if information related to different events were to be inappropriately combined [66, 67]. What remains critically unknown is how age-related changes in combining visual and vestibular information might affect the perception of self-motion. Here, we have identified an increased sensitivity to visually induced self-motion with increasing age, which may be compensating for a potential age-related decline of the vestibular system [68, 69]. Our results suggest that the efficacy of vection increases with age, consistent with Paige’s finding for circular vection [56]. In conflict with this conclusion, Shirai et al. [4, 8, 9] reported that vection magnitude and saturation decreased with age from childhood to young adulthood. It is likely that this difference is because visual odometry reflects the direct accumulation of self-motion information and is probably unrelated to conventional vection magnitude scales [12].

As children grow, their sensory systems must adapt to changes such as an increase in the separation of the eyes which is a key calibrating factor determining the relationship between the distance of a target and the convergence of the eyes [70]. These changes in interocular distance (IOD) are most substantial in infancy but there would likely be several millimetres difference between the average IOD of our youngest and oldest participants [71]. Our HMD assumes a fixed IOD. This means that participants with different IODs may have had different amounts of vergence while looking at a given target. This might be expected to influence space perception but any such differences cannot explain the age effect we found because (1) any such helmet-induced distance distortions would affect the scene equally during both target presentation and the simulated motion, (2) vergence cues are weak for the target distances we used (all our targets were 10m or more away) and observers have a surprising perceptual tolerance for errors in IOD used in image generation in realistic scenes, and (3) the age effect was not restricted to childhood where variation in IOD with age would be most pronounced.

### Citizen science

**Fig 9:**
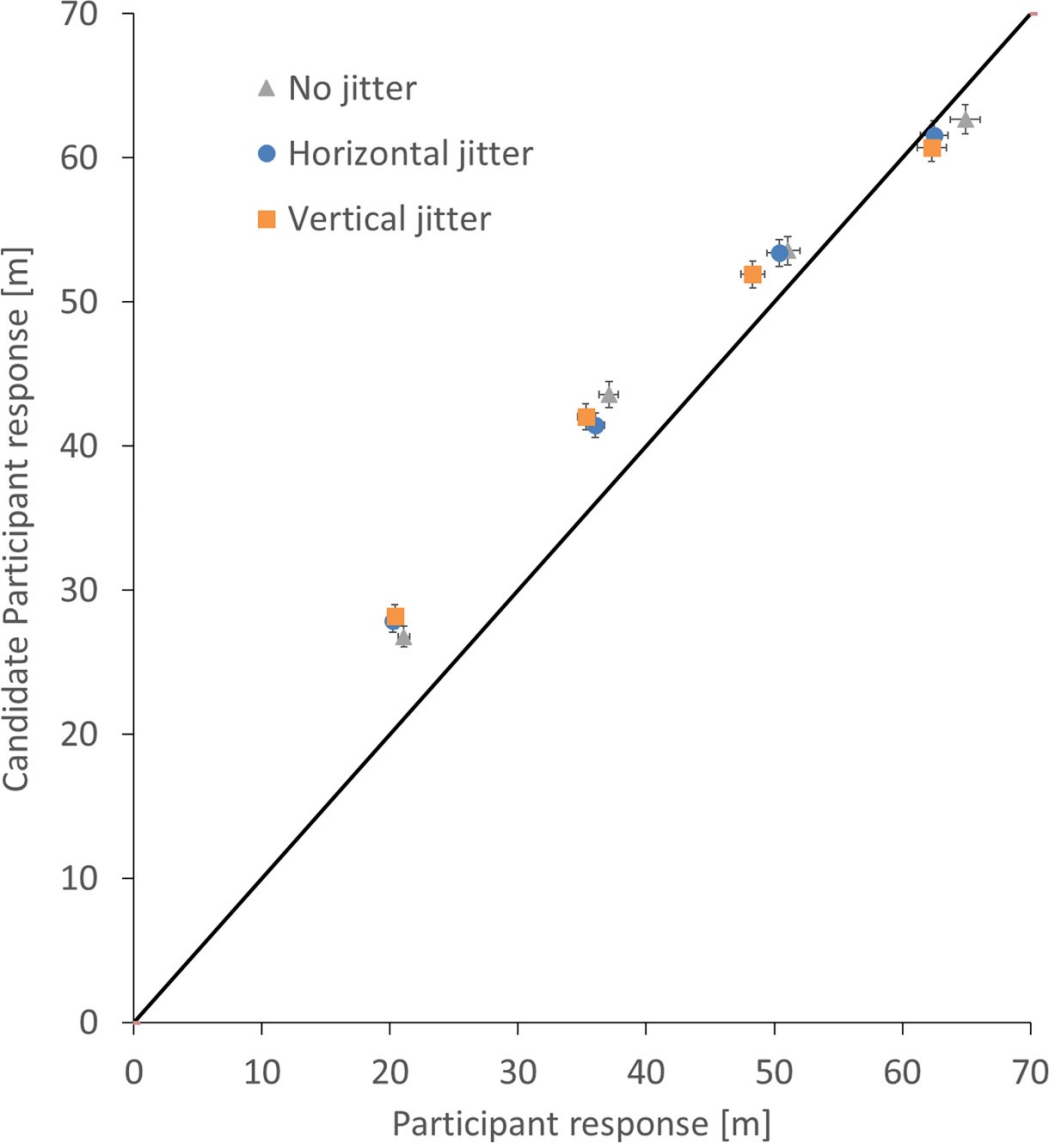
The mean of candidate participants’ responses plotted against the mean of participants’ responses. The mean response distance for each of the four target distances of the candidate participants (n = 871) plotted against the participants’ mean distance (n = 466). Data from the three jitter conditions are plotted separately. The solid line indicates a slope of one, where both populations are the same. Error bars show standard errors in both the participants’ responses (horizontal) and the candidate participants’ response (vertical).

This experiment was conducted at the Ontario Science Centre and participation was open to all visitors to the centre. Citizen science is very rewarding activity both for science and for the participants. From the scientist’s perspective citizen science provides us with a large sample size where significant effects can be detected despite small effect sizes. Moreover, the results will be more easily generalizable than when data are collected from a small group of university-age students. From the participant’s perspective, the opportunity to take part in experiments that are designed to advance science provides an educational, meaningful, and enjoyable experience. Citizen science demonstrates the scientific method to many people of all ages, most of whom have probably never taken part in an experiment before. However, citizen science also has some potential drawbacks. Not all participants will be equally invested in completing an actual experiment, no matter how trimmed down the experimental conditions might be, and no matter how engaging the experiment. Dealing with the ‘tail ends’ of the distribution of data obtained in citizen science experiments is a potential problem; public participants can be ‘overly eager’ or ‘generally disinterested’ in a study, thus skewing the data in one way or another. Our experiment at the Ontario Science Centre was not specifically advertised in advance to the general public, and so we did not anticipate having many overly eager participants. But we were aware of potentially having some generally disinterested participants or people, particularly children, who got distracted by the lollipop world and responded in a manner that was more or less independent of the stimuli being presented. In order to accommodate these potential biases, we implemented reasonably strong selection criteria for inclusion in the participant pool, resulting in a 47% attrition rate in terms of participants to participants. One question that naturally arises with such a high attrition rate, is what did the performance of the rejected participants look like? This is addressed in Fig 9 which plots mean accepted participant responses on the horizontal axis against mean rejected participant responses on the vertical axis (see methods for acceptance criteria). If the two groups responded in a similar manner, all points would lie on a line with a slope of one. However, the rejected participants generally pressed the button later, consistent with them not paying attention to the task. Table 2 summarizes the effect of the various criteria in terms of selecting participants from the candidate participants. Having strong and easily computable criteria to separate less invested from more invested participants is an important part of the design of citizen science experiments.

## Conclusions

This experiment was carried out in a public environment involving a large number of people of all ages, each providing a small amount of data. By applying rigid acceptance criteria, we were able to obtain reliable data from participants as young as four years old. We showed how, although there are individual differences, on average participants had higher perceptual gains and needed less visual motion to evoke the perception of having travelled through a particular distance in the presence of vertical jitter. The effectiveness of vection was found to increase with age over all jitter conditions (perceptual gain increased with age) and the onset delay of vection (calculated from the intercepts of the linear fits) increased slightly with age. There were no differences between the sexes. Our study suggests that a greater amount of visual movement should be provided when simulating self-movement in virtual reality for younger participants but that this should be toned down for older adults. Citizen science studies like this can provide a unique and valuable insight into perceptual processes in a truly representative sample of people.
